# Implementation of an English and Spanish Dementia Screening Toolkit in Primary Care

**DOI:** 10.1093/geroni/igaf122.1097

**Published:** 2025-12-31

**Authors:** Mirella Diaz-Santos, Gabriela Islas Huertas, Samantha Shah, Satpal Singh Wadhwa, Blanca Campos, Marjan Dehghanian, Michelle Bholat, Timothy Chang

**Affiliations:** University of California, Los Angeles, Los Angeles, California, United States; David Geffen School of Medicine at UCLA, Los Angeles, California, United States; David Geffen School of Medicine at UCLA, Los Angeles, California, United States; David Geffen School of Medicine at UCLA, Los Angeles, California, United States; University of California, Los Angeles Department of Family Medicine, Los Angeles, California, United States; University of California, Los Angeles Department of Family Medicine, Los Angeles, California, United States; University of California, Los Angeles Department of Family Medicine, Los Angeles, California, United States; David Geffen School of Medicine at UCLA, Los Angeles, California, United States

## Abstract

**Introduction:**

Dementia affects over 10% of individuals aged 65 and older, with Black and Hispanic/Latinos experiencing a 1.5-2.0 times higher prevalence than non-Hispanic Whites. Despite the importance of early detection, adoption of dementia screeners in primary care is challenging. This study presents a primary care champion intervention focused on providers’ adoption of a brief dementia screening toolkit (DST).

**Methods:**

Intervention implemented strategies to assist physicians, and staff in the integration of the electronic DST into their routine clinical care. The DST included a three-item questionnaire and a cognitive assessment (Mini- Cog). Primary care providers recruited patients aged 60+ to complete the DST during their annual wellness visits. We compared providers’ adoption of the two DST components, pre-intervention (September 2022-March 2023), and post-primary care champion intervention (April 2023-February 2024).

**Results:**

Seven out of 45 providers engaged with the DST pre-intervention, and 39 providers post-intervention. Providers enrolled 781 and 1,329 patients pre-and post-intervention, respectively. Mean age of patients was 70 (range: 60–103). Recruited patients mirrored the clinic’s demographics: 80% Non-Hispanic Whites, 10% Asian/Asian Americans and 10% Black/African Americans, and 31% Hispanics. Pre-intervention, 60% completed the questionnaire while 5.3% completed the Mini-Cog. 57.1% and 26.3% completion were obtained post-intervention. Pre-post questionnaire completion analyses showed no significance (OR 1.14, p = 0.42). Completed Mini-Cogs significantly increased by 375% (OR 6.6, p < 0.0001).

**Discussion:**

Results underscore the impact of implementing a primary care champion intervention to improve providers’ adoption of dementia screeners with a cognitive assessment into routine clinical care.

